# A Pilot Randomised Controlled Trial of a Targeted Dietary Approach to Potassium Management in Hemodialysis: A Clinical Research Protocol for the Evidence for Optimal Potassium Intake in Hemodialysis (EvoKe-HD) Study

**DOI:** 10.1177/20543581261476635

**Published:** 2026-08-19

**Authors:** Amélie Bernier-Jean, Rémi Goupil, François Madore, Jessica Dawson, Roxanne Papineau, Allison Jaure, Catherine Clase

**Affiliations:** 1 67120 Centre de recherche, CIUSSS du Nord-de-l’Île-de-Montréal, Montreal, QC, Canada; 2 Faculté de médecine, 5622 Université de Montréal, Montreal, QC, Canada; 3 110588 NHMRC Clinical Trials Centre, Camperdown, NSW, Australia; 4 55973 Institut universitaire de cardiologie et de pneumologie de Québec, Quebec, QC, Canada; 5 Faculty of Medicine and Health, 117637 The University of Sydney School of Public Health, Sydney, NSW, Australia; 6 62703 McMaster University Faculty of Health Sciences, Hamilton, ON, Canada

**Keywords:** hemodialysis, hyperkalemia, dietary potassium, potassium additives, pilot study

## Abstract

**Background:**

Hyperkalemia affects more than one in five patients receiving maintenance hemodialysis and remains a major contributor to arrhythmias and sudden cardiac death. Traditional potassium restriction has centered on limiting fruits and vegetables, even as emerging evidence challenges the assumption that all high-potassium foods pose equal risk. Meanwhile, awareness of potassium additives has grown, and recommendations to avoid additive-containing processed foods have been layered onto existing restrictions. Together, the uncertainties around long-standing to newer recommendations create guidance that is difficult for many patients to navigate.

**Objective:**

To describe the protocol for EvoKe-HD, a pilot randomised controlled trial evaluating the feasibility, acceptability and safety of a targeted dietary counselling strategy for potassium management in hemodialysis.

**Design:**

Two-arm, 1:1 randomised, parallel-group feasibility trial.

**Setting:**

Two dialysis units at the CIUSSS du Nord-de-l’Île-de-Montréal, Canada.

**Patients:**

Adults receiving thrice-weekly maintenance hemodialysis with elevated pre-dialysis serum potassium levels or receiving regular potassium binder therapy.

**Measurements:**

The primary feasibility outcome is the proportion of eligible patients who enrol and complete the 3-month assessment. The main exploratory clinical outcome is mean mid-week pre-dialysis serum potassium during follow-up. Secondary and exploratory outcomes include hyperkalemia events, potassium binder use, dialysate potassium adjustments, biochemical measures, dietary outcomes, patient-reported outcomes, and process evaluation measures.

**Methods:**

Participants are randomised to monthly targeted dietary counselling focused on reducing potassium additives and selected high-impact foods, or to monthly standard potassium-restriction counselling based primarily on total potassium content. Feasibility outcomes will be summarized with 95% confidence intervals. Mean follow-up serum potassium will be analysed using linear mixed-effects model adjusted for baseline potassium and sex, with a random intercept for participant.

**Results:**

Recruitment began in June 2025 and was completed in January 2026. The last study visit was completed in March 2026. Process evaluation interviews are expected to be completed in July 2026.

**Limitations:**

As a single-centre pilot feasibility trial, EvoKe-HD is not powered to evaluate definitive effects on serum potassium, hyperkalemia events, or other clinical outcomes.

**Conclusions:**

EvoKe-HD will assess whether a targeted potassium counselling strategy can be implemented in routine hemodialysis care and will provide feasibility and process evaluation data to inform the design of a larger trial.

## Introduction

Hyperkalemia affects approximately 22% of patients receiving maintenance hemodialysis and remains a major contributor to arrhythmias and sudden cardiac death.^1,2^ For decades, its management has relied largely on dietary restriction, with a heavy focus on avoiding high intakes of fruits and vegetables and plant protein sources such as legumes, nuts and seeds. Recent evidence suggests, however, that not all dietary potassium sources contribute equally to serum potassium levels. Many whole plant foods appear to result in a lesser increase in serum potassium levels than their total content would suggest, likely because of differences in food structure and fibre content, although this varies across foods.^3-6^ In contrast, potassium from additives, juices, processed foods, and many animal products appears to result in near-complete systemic potassium availability.^3^

As awareness of the potassium contribution from additives has grown,^7^ clinical practice has gradually layered advice to avoid additive-containing processed foods on top of longstanding restrictions targeting fruits and vegetables. The cumulative effect is a dietary approach that is increasingly restrictive and difficult to implement, despite limited evidence that this overall counselling strategy meaningfully improves potassium control in routine practice. Clinicians and dietitians increasingly agree that potassium additives and certain processed foods may play a disproportionate role in hyperkalemia, yet current recommendations still rely heavily on generalized food lists rather than evidence-driven prioritization.^8^

Despite decades of dietary guidance in dialysis care, very few studies have tested real-world counselling strategies that reflect how dietary advice is actually delivered in routine dialysis care, and none have evaluated an approach that explicitly prioritizes potassium additives. Feeding studies and nutrient composition analyses do not reflect the complexity of routine counselling, patient preferences, or the practical challenges of navigating multiple overlapping dietary restrictions. As a result, the field lacks empirical evidence to guide a targeted, patient-centered potassium strategy.

This has led to interest in a more focused approach that limits foods most likely to raise serum potassium while avoiding unnecessary restriction of nutrient-dense plant foods. EvoKe-HD is a pilot randomised controlled trial designed to evaluate the feasibility, acceptability, safety and implementation of such a targeted dietary strategy, centered on reducing potassium additives and other foods believed to disproportionately increase serum potassium levels rather than broadly restricting all high-potassium foods.

## Methods

### Study Design

EvoKe-HD is a two-parallel-group, 1:1 randomised, feasibility, controlled trial.

### Study Setting

The study will be conducted at the dialysis facilities of the CIUSSS du Nord-de-l’Île-de-Montréal which comprise two outpatient dialysis clinics, one in-centre dialysis unit and one semi-autonomous off-centre dialysis unit.

### Eligibility Criteria

Inclusion criteria:

• 18 years or older
• Receiving hemodialysis 3 times/week for ≥ 3 months
• Has at least two pre-dialysis serum potassium levels ≥5.2 mmol/L in the past 3 months or has a most recent pre-dialysis serum potassium value ≥4.6 mmol/L while currently receiving regular potassium binder therapy (≥ once per week).
• Can speak and understand French or English
• Has not missed more than 1 dialysis session in the previous 3 months.
• Is capable of providing informed consent.

Exclusion criteria:

• Is not expected to survive beyond 6 months
• Does not control their dietary intake (e.g., residents of high-dependency nursing homes)
• Significant cognitive impairment precluding understanding of the dietary recommendations.

Participants are not required to consume potassium additives at baseline to be eligible. The intervention is individualized so that counselling focuses on the participant’s actual dietary potassium sources identified through recall.

### Experimental Intervention (See Table 1)

#### First Session (Baseline)

Participants randomised to the experimental group will receive an initial individual dietary counselling session with the research dietitian, conducted in person (Table 1). During this session, the dietitian will meet with the participant in person and complete a 24-hour dietary recall using ASA24-Canada2018 and provide structured education on:

• the role of potassium in the body
• the risks associated with elevated potassium levels
• why individuals with kidney disease are more prone to hyperkalemia
• the concept that different foods contribute differently to serum potassium levels
• the dietary sources believed to contribute most substantially, including potassium additives.

**Table 1.** Major Differences Between the Proposed and the Traditional Approach to Potassium Dietary Counselling in Hemodialysis

​	Proposed approach	Standard of care
Focus of restriction	Additives and other food sources believed to contribute disproportionately to serum potassium levels	Foods with high total potassium content
Whole fruits and vegetables	Not discouraged	Intake of moderate to high potassium fruits and vegetables is restricted
Potassium additives	Education on identifying and avoiding four specific potassium additives	Potassium additives are listed in the participant materials but are not emphasized relative to other high-potassium foods
Materials provided	One double-sided handout focused on identifying potassium additives and avoiding foods believed to contribute disproportionately to serum potassium levels	List of foods with moderate and high total potassium content

Participants will then be taught to identify and avoid four potassium additives (potassium chloride, potassium phosphates, potassium citrate, and potassium lactate) based on the highest-risk classification by Picard et al. Each participant, in consultation with the study dietitian, will then select two additional foods from a predefined list (meats, dairy products, potatoes, fruit juices, sugary drinks, coffee, alcohol, chocolate, maple syrup, molasses) that they will attempt to reduce. Reductions in nutritious foods (e.g., meat or dairy) are recommended only when intake is excessive and judged to contribute to hyperkalemia, and never at the expense of overall nutritional adequacy. Where reductions conflict with other nutrition priorities, the dietitian will propose alternatives. All recommendations will be individualized.

Therefore, both groups receive active potassium counselling from a renal dietitian. The trial does not compare advice versus no advice, nor additive avoidance versus additive permission. Rather, it compares two ways of prioritizing dietary potassium counselling:

(1) a targeted strategy that emphasizes additives and selected high-impact foods while avoiding unnecessary restriction of nutrient-dense plant foods, and
(2) contemporary standard counselling based primarily on total potassium content, in which additives are included but not specifically prioritized.

Participant-facing materials were developed using established health literacy principles, informed by the Patient Education Materials Assessment Tool,^9^ and reviewed by two patient partners to ensure clarity, relevance, and acceptability. Participant-facing materials will be available in French and English (the English version is available in the Supplementary Material).

#### Subsequent Sessions (Monthly)

Follow-up sessions will be conducted in person whenever possible, or by telephone when necessary. At each session, the dietitian will complete a 24-hour dietary recall using ASA24-Canada2018, review progress toward the actions selected at the previous visit, reinforce successes, and discuss barriers. If pre-dialysis serum potassium remains high, the dietitian will suggest additional actions from the same predefined list used during the initial intervention.

### Control Group

Participants randomised to the control group will also receive monthly individual dietary counselling sessions with the research dietitian.

#### First Session (Baseline)

The baseline session will be conducted in person. The dietitian will complete a 24-hour dietary recall using ASA24-Canada2018 and will provide structured education on potassium physiology, the risks of hyperkalemia, and reasons for increased susceptibility in kidney disease. Counselling will then be based on the standard dietary reference used in our centre (Guide d’alimentation pour les personnes dialysées, 2nd Edition), which outlines foods with moderate to high potassium content. Participants will receive these lists in French or English.

During study preparation, this guide was updated to include potassium additives and foods newly classified as very high in potassium. To ensure the control arm reflects usual care, this revised version will be used;, but additives will not be emphasized more than other high-potassium foods. This comparator was chosen because it reflects current real-world evolution in potassium counselling at our centre. 

#### Subsequent Sessions (Monthly)

At each follow-up visit (conducted in person whenever feasible, or by telephone otherwise) the dietitian will complete a 24-hour dietary recall using ASA24-Canada2018, review progress toward previously selected actions, reinforce successes, and discuss challenges.

### Medication and Dialysis Prescription

As a pragmatic pilot trial embedded in routine dialysis care, co-interventions such as potassium binders and dialysate potassium adjustments will remain at clinician discretion. These will be captured systematically because they are part of real-world hyperkalemia management and are relevant for assessing the feasibility and interpretability of a larger definitive trial. To avoid conflicting dietary messages, physicians, nurses, and other health professionals will be asked to refrain from providing potassium-related dietary advice to study participants during the trial.

### Other Dietary Interventions

Interventions targeting other aspects of nutrition, such as caloric intake, protein intake, and phosphorus management, will continue as per usual care. At the participating dialysis units, there is currently no systematic nutritional evaluation by a renal dietitian, these assessments occur at the request of the treating physician. While physicians will be asked not to request a nutritional evaluation specifically for hyperkalemia for the duration of study, they may request dietitian involvement for any other indication.

### Criteria for Discontinuing or Modifying the Intervention

The dietary intervention will be temporarily suspended during hospitalisation and discontinued if a participant receives a kidney transplant or permanently transitions off maintenance hemodialysis. Participants may also discontinue the dietary intervention at any time upon request. All modifications or discontinuations of the intervention will be documented.

### Outcomes

The primary feasibility outcome will be the proportion of eligible patients who consent to participate and complete the 3-month assessment. The main exploratory clinical outcome will be the average mid-week pre-dialysis serum potassium level across all routine monthly assessments performed during the 3-month intervention period. Repeat measurements obtained specifically to confirm or follow up an elevated potassium value will be excluded to avoid overrepresentation of clinically triggered measurements that would bias estimates. This outcome will provide exploratory clinical data to inform whether and how serum potassium should be evaluated in a larger trial. Tables 2 and Supplementary Table S1 list the secondary feasibility outcomes and exploratory clinical and safety outcomes, respectively. Other secondary outcomes will include the Subjective Global Assessment (SGA), the Standardised Outcomes In Nephrology (SONG)-Fatigue measure, a patient-reported measure of satisfaction with food,^10^ albumin, calcium, phosphorus and parathyroid hormone (PTH) levels, blood pressure, interdialytic weight gain and the content of the dietary assessments (Supplementary Table S2). All secondary outcomes will be exploratory and not powered for between-group differences.

**Table 2.** Feasibility Outcomes

​	Low	Probable	High
% of eligible patients who consent to participate and complete the 3-month assessment*	<30	30-40	>40
% of screened participants randomised	<40	40-80	>80
Number of participants recruited 12 months into the trial	<30	30-40	>40
% of randomized participants who report having understood the dietary recommendations well or very well using a questionnaire	<40	40-60	>60
% of randomized participants who judge the intervention to be acceptable using a questionnaire	<50	50-60	>60

*Primary feasibility outcome.

### Sample Size

Detailed assumptions and calculations are provided in the Supplementary Material. For this pilot study, we aim to recruit up to 60 participants and retain approximately 50, which will allow us to estimate the primary feasibility outcome with acceptable precision (±14%) and provide exploratory clinical estimates for mean mid-week pre-dialysis serum potassium during follow-up.

### Recruitment

Research personnel will proceed to a first screen of all the prevalent individuals receiving dialysis treatments at our center, selecting those with two or more serum potassium measurements ≥5.2 mmol/L or ≥4.6 mmol/L with binders in the past 3 months, using the blood test result software of the institution. The identified potential participants will then be further assessed for the remaining eligibility criteria. A screening log will be kept listing all patients that were assessed for enrolment and where relevant, the reason for exclusion.

### Allocation

#### Sequence Generation

Participants will be randomised 1:1 using a computer-generated random sequence to intervention or control, stratifying for gender since food procurement and preparation roles tend to be distributed differentially across genders.

#### Allocation Concealment Mechanism

The randomisation sequence will be implemented in REDCap which conceals it from the research personnel and investigators.

### Blinding

Due to the nature of the intervention, participants, the study coordinator and the dietitian providing the intervention will not be blinded to sequence allocation. The investigators, and the dialysis physicians and personnel will remain blinded to the group allocation.

### Data Collection Methods

All participants will be followed at their scheduled dialysis sessions to minimize loss to follow-up. Data collection will occur monthly (Supplementary Table S3).

#### 24-hour Dietary Recalls

Monthly dietary assessments will use the 24-hour dietary recall using the U.S. Department of Agriculture’s (USDA) Automated Multiple-Pass Method (version ASA24-Canada2018 available in French and English) which is widely used in nutritional studies and well validated.^11,12^ 24-hour dietary recalls are highly correlated with weighted food records in the dialysis population,^13^ use structured prompts which will allow us to capture potential sources of potassium additives and can account for cooking methods.

Repeated 24-hour recalls have better accuracy than single assessment.^14^ For this reason, two non-consecutive 24-hour dietary recalls will be conducted at baseline and at 3 months. The second dietary recall will be conducted within one week prior to the meeting with the dietitian. To reduce biases arising from the participants’ level of literacy and comfort with technology, the dietitian will complete the online recall with the participant using a portable device.

#### Serum Potassium Collection

Serum potassium values will be obtained from routine clinical blood draws performed before the mid-week dialysis session and analysed by the institutional laboratory using standard operating procedures. Serum potassium will be analysed by ion-selective electrode potentiometry in the hospital clinical biochemistry laboratory.

### Statistical Methods

The primary feasibility outcome, the proportion of eligible patients who enrol and complete the study, will be summarized with a 95% confidence interval. Recruitment, randomisation, retention, understanding of dietary recommendations, and acceptability outcomes will be interpreted using the prespecified feasibility thresholds shown in Table 2.

The main exploratory clinical outcome of mean mid-week pre-dialysis serum potassium during follow-up will be analysed using a linear mixed-effects model. The model will include fixed effects for treatment group, time, gender as the stratification factor, and baseline serum potassium, defined as the mean of the two pre-randomisation potassium measurements. A random intercept will be included to account for the correlation between repeated post-randomisation potassium measurements from the same participant. Routine monthly mid-week pre-dialysis potassium measurements obtained during the 3-month intervention period will be included, whereas repeat measurements obtained specifically to confirm or follow up an elevated potassium value will be excluded to avoid overrepresentation of clinically triggered measurements.

Analyses will follow the intention-to-treat principle and will include all randomised participants with at least one post-baseline potassium measurement. All other clinical outcomes will be summarized descriptively without formal hypothesis testing.

### Process Evaluation

A process evaluation will be conducted in accordance with the UK Medical Research Council guidance^15^ to examine the implementation, acceptability, and potential scalability of the intervention. Process evaluation findings will be used to refine the intervention manual for the larger trial.

Pre- and post-intervention questionnaires will assess participants’ understanding of the dietary recommendations, their ability to translate them into meaningful changes, their perception of the acceptability of the proposed modifications, and their overall experience of the counselling provided by the dietitian.

Participants from the intervention group and study staff (nurses, nephrologists, and dietitians) will be selected using purposive sampling to capture diversity in demographic and clinical characteristics. Semi-structured interviews will explore barriers and facilitators to acceptance and uptake of the intervention, perceptions of fidelity and quality of implementation, and contextual factors that may influence variation in outcomes. Interviews will be conducted by a member of the research team who was not involved in recruitment or intervention delivery. All interviews will be audio recorded and transcribed verbatim. Data will be analysed using a framework-based qualitative approach informed by the UK Medical Research Council process evaluation guidance. Interviews will continue until data saturation is reached. Quantitative and qualitative findings will be triangulated to inform refinement of the intervention.

### Adverse Events

Spontaneously reported adverse events and unintended effects of the interventions will be documented throughout the trial. In addition, adverse events and hospitalizations will be collected systematically at the end of the intervention through review of the participant’s medical record, regardless of whether they are judged to be related to the intervention. An independent committee will classify each event as probably, possibly, unlikely, or not related to the intervention.

## Results

This manuscript is based on protocol version 7.1 dated 19 January 2026. Ethics approval was obtained on 18 April 2024 from the Comité d’éthique de la recherche du CIUSSS du Nord-de-l’Île-de-Montréal (Ref No. 2024-2643). Recruitment began in June 2025 and was completed in January 2026. The trial enrolled 50 participants. Recruitment was stopped at this point because attrition was lower than anticipated and the enrolled sample size was considered sufficient to meet the objectives of this pilot feasibility trial. The last study visit was completed in March 2026. Process evaluation qualitative interviews are ongoing and are expected to be completed in July 2026. Detailed feasibility outcomes, including recruitment and retention outcomes, will be reported with the main trial results.

## Protocol Amendments

During the trial, two protocol amendments were introduced. First, the intervention duration was shortened from 6 to 3 months, as it became apparent early in the trial that a 3-month follow-up was sufficient to address the primary feasibility objectives. Second, an additional inclusion criterion was added to better capture patients with elevated serum potassium managed with pharmacological therapy: pre-dialysis serum potassium ≥4.6 mmol/L while receiving regular potassium binder therapy (≥ once per week). Both amendments were approved by the research ethics board prior to implementation.

## Conclusions

EvoKe-HD addresses an emerging evidence gap in dietary potassium management by testing whether a targeted potassium counselling strategy can be implemented in routine hemodialysis care. As guidance moves away from blanket restriction of potassium-rich whole foods, there remains little pragmatic evidence on how to prioritize potassium additives and selected high-impact foods while preserving overall diet quality and patient acceptability. This pilot trial will estimate recruitment and retention, assess acceptability and implementation, and generate exploratory clinical data on serum potassium and related outcomes. These findings will inform whether and how to design a larger trial of targeted, nutritionally protective potassium counselling in hemodialysis.

## Supplemental Material

Supplemental Material - A Pilot Randomised Controlled Trial of a Targeted Dietary Approach to Potassium Management in Hemodialysis: A Clinical Research Protocol for the Evidence for Optimal Potassium Intake in Hemodialysis (EvoKe-HD) Study Supplemental Material for A Pilot Randomised Controlled Trial of a Targeted Dietary Approach to Potassium Management in Hemodialysis: A Clinical Research Protocol for the Evidence for Optimal Potassium Intake in Hemodialysis (EvoKe-HD) Study by Amélie Bernier-Jean, Rémi Goupil, François Madore, Jessica Dawson, Roxanne Papineau, Allison Jaure, Catherine Clase in Canadian Journal of Kidney Health and Disease

## Data Availability

Data generated during the EvoKe-HD study will not be publicly available. The creation of a research database for secondary use and data sharing was not included in the original ethics approval, and any data sharing would require additional approval from the Research Ethics Committee.*
