# Supplementary material for: A Pilot Randomised Controlled Trial of a Targeted Dietary Approach to Potassium Management in Hemodialysis: A Clinical Research Protocol for the Evidence for Optimal Potassium Intake in Hemodialysis (EvoKe-HD) Study
Source: Can J Kidney Health Dis. 2026 Aug 19;13:20543581261476635. doi: 10.1177/20543581261476635 (PMC13490776; doi:10.1177/20543581261476635)
Supplement: Supplementary file 1 [file sj-pdf-1-cjk-10.1177_20543581261476635.pdf]

Table of Contents for the Supplement

Supplementary Methods: Sample Size Calculation 2

Table S1. Exploratory efficacy and safety outcomes 4

Table S2. Other outcomes 5

Table S3. SPIRIT Participant Timeline 6

SPIRIT Checklist 7

Participant Material – Intervention group 9

References 11

Supplementary Methods: Sample Size Calculation

The primary outcome of the planned larger EvoKe-HD trial will be time to first hyperkalemia event, censored for death or kidney transplantation. To achieve 80% power to detect a hazard ratio of 0.70 (corresponding to a 30% relative reduction) at a two-sided type I error rate of 0.05, a total of 247 primary events would be required.

Based on data from our local eligible population, the distribution of hyperkalemia events is highly skewed, with a small proportion of patients experiencing multiple events. The median number of hyperkalemia events is approximately 2.6 per person-year, indicating that first events occur frequently in this population. Assuming a constant annual censoring rate of 30% due to death or kidney transplantation, we estimate that approximately 284 participants followed for 2 years would be required to accrue the target number of events.

Leveraging our network of collaborators, we anticipate that recruiting from six dialysis sites of size similar to our center (approximately 47 participants per site; total 282 participants) would be feasible for the larger trial. The primary objective of the present pilot study is therefore to estimate key feasibility parameters, including recruitment and retention rates, to inform the planning and scalability of this multicentre trial.

Recruiting 60 participants and retaining 50 at the end of the 3-month pilot intervention would allow estimation of the primary feasibility outcome with a precision of ±14%.

Although the planned larger EvoKe-HD trial will evaluate time to first hyperkalemia event, this pilot trial is also intended to provide proof-of-concept evidence based on mean mid-week pre-dialysis serum potassium during follow-up. Assuming 25 participants per group and approximately six post-randomization potassium measurements per participant, and using

variance components estimated from routine-care potassium data over a comparable 12-week period (random-intercept SD 0.48 mmol/L; residual SD 0.56 mmol/L; ICC 0.42), the minimal detectable difference in mean follow-up potassium is approximately 0.42 mmol/L for 80% power at a two-sided α of 0.05 when analysed using a linear mixed effects model with a random intercept.

Adjustment for baseline potassium, defined as the mean of two pre-randomization measurements, and sex (the stratification factor) is expected to further increase precision, depending on the correlation between baseline and follow-up potassium. Observational studies have shown an increased risk of all-cause mortality with mid-week pre-dialysis serum potassium ≥5.6 mmol/L compared with a reference range of 5.0 to 5.29 mmol/L.[1] Therefore, a between-group difference of approximately 0.4 mmol/L in average serum potassium is considered clinically meaningful.

Table S1. Exploratory efficacy and safety outcomes

Specific outcome	Rationale
*Average mid-week pre-dialysis serum potassium level across all routine monthly assessments performed during the 3-month intervention period **	High 3-month average serum potassium was associated with increased all-cause mortality[1-3]
Peak serum potassium over the study period<br><br>Number of hyperkalemia (≥5.6 mmol/L) and severe hyperkalemia (>6 mmol/L) events	Peak serum potassium was associated with increased short-term mortality in hemodialysis[4]
Number of dialysis sessions with dialysate concentration < 2 mmol/L<br><br>Number of patients for whom the potassium dialysate concentration was raised or lowered (by ≥ 0.5mmol/L)	Circumstantial evidences suggest an increased risk of cardiac events with potassium dialysate < 2 me/L[5]
Total dose of potassium binders and number of participants requiring potassium binder at 3 months	Potassium binders use may be associated with fatal gastrointestinal injury [6] and may affect appetite and quality of life
Measures of diet quality (healthy diet index, Mediterranean diet score, fiber intake, ratio of plant to animal protein intake, daily intake of fruits and vegetables, etc.)	The interventions may impact the participant's dietary patterns.
Number of servings/day of intervention-targeted high potassium items (meat, dairy, juices, potatoes…)<br><br>Number of servings/day of moderate to high total potassium food items<br><br>*"I have changed my diet according to the dietitian's recommendations."* (1-7 Likert scale with anchors)<br><br>*"I follow the diet recommended by the dietitian."* (1-7 Likert scale with anchors)	Assesses whether the intervention was successful at modifying the intake of potassium sources.

*main exploratory efficacy and safety outcome

Table S2. Other outcomes

Category	Specific outcome	Rationale
**SONG-HD core outcomes**	Mortality, cardiovascular disease (sudden cardiac death, MI, adjudicated by an independent physician), and fatigue (SONG fatigue measure)	Outcomes identified by patients, caregivers, and health professionals as critical to all trials in hemodialysis[7]
**Mineral and bone disorder**	Average monthly midweek pre-dialysis serum phosphate, calcium and parathyroid hormone levels during the study period	Consuming less processed food may reduce phosphorus intake[8-13]
**Volume status**	Short-dialytic interval weight gain<br><br>Pre dialysis blood pressure	Consuming less processed food may reduce sodium intake, reducing thirst and fluid intake[14, 15]
**Acid-base status**	Average serum bicarbonate levels	Diet composition can affect bicarbonate levels[13, 16, 17]
**Satisfaction with food**	5-item scale: Cronbach's alpha 0.81, 6-month test-retest reliability 0.6, correlations of 0.26 – 0.36 with measures of global quality of life.[18]	Intervention expected to be less restrictive and more enjoyable than the traditional dietary approach
**Nutritional status**	Subjective global assessment<br><br>Albumin and prealbumin levels, normalized protein nitrogen appearance (calculated from urea reduction ratio), creatinine and body mass index	Adverse events assessment

Table S3. SPIRIT Participant Timeline

Phase	Enrollment	Baseline	Months 1	Months 2	Month 3
**Enrollment**					
Eligibility screening	•				
Informed consent	•				
Allocation (randomisation)	•				
**Interventions**					
Experimental counselling		•	•	•	
Standard counselling (control)		•	•	•	
**Assessments**					
Demographics, medical history	•				
Clinical: weight, BP, dialysis parameters		•	•	•	•
Serum biochemistry (incl. potassium, phosphate, calcium, HCO_3)		•	•	•	•
Urine 24h collection for potassium, urea and creatinine	•				
Estimated stool volume		•			
Medication (including potassium and phosphate binders)		•	•	•	•
Satisfaction with food-related life		•	•	•	•
24h dietary recall (completed on an electronic tablet by the dietitian)	•	•	•	•	•
Subjective Global Assessment		•			•
Adverse events		•	•	•	•

SPIRIT Checklist

Item	Description	Page no.(s)
1	Descriptive title identifying study design, population, interventions, and acronym	1
2a	Trial registration identifier and registry name	1
2b	WHO Trial Registration Data Set items	1
3	Protocol version number and date	1
4	Funding sources and type of support	14
5a	Names, affiliations, and roles of contributors	1, 15
5b	Name and contact information for sponsor	1
5c	Role of funders	14
6a	Background and rationale	4–5
6b	Explanation for choice of comparators	4–5, 7–8
7	Specific objectives or hypotheses	5
8	Trial design	5
9	Study setting	5
10	Eligibility criteria	5–6
11a	Interventions for each group	6–8
11b	Criteria for modifying or discontinuing interventions	9
11c	Strategies to improve adherence	7-8, 12
11d	Relevant concomitant care	9
12	Outcomes	9–10
13	Participant timeline	Supplementary Table S3
14	Sample size	10
15	Recruitment	10
16a	Sequence generation	11

16b	Allocation concealment	11
16c	Implementation	11
17a	Blinding	11
17b	Unblinding	N/A
18a	Data collection methods	11–12
18b	Data quality	11–12
19	Data management	14
20a	Statistical methods	12
20b	Additional analyses	12
20c	Missing data	12
21a	Data monitoring committee	N/A
21b	Interim analyses	N/A
22	Harms	13
23	Auditing	N/A
24	Ethics approval	14
25	Protocol amendments	14
26a	Informed consent	14
26b	Additional consent	N/A
27	Confidentiality	14
28	Declaration of interests	14
29	Access to data	14
30	Ancillary care	N/A
31a	Dissemination policy	14
31b	Authorship	1
31c	Public access to protocol	1

Participant Material – Intervention group

My potassium is high. What should I do ?

Before eating packaged foods, check the ingredients list and look for anything with the word **POTASSIUM.**

Avoid foods containing any of the following ingredients :

- **Potassium chloride**
- **Potassium phosphates**
- **Potassium citrate**
- **Potassium lactate**

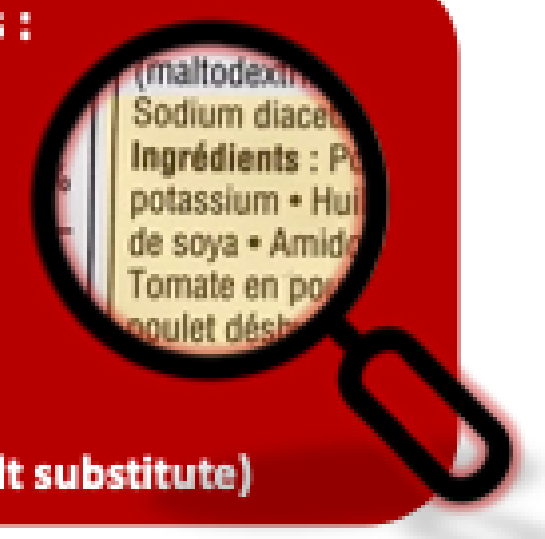

Avoid potassium-based salt substitutes
(examples: Windsor's "No Salt" or "Half Salt" or Herbamare's Salt substitute)

Why? Because your body will absorb 100% of the potassium contained in these additives.

With the help of your dietitian, select 2 actions from the following list:

☐ Reduce your consumption of **animal protein** to ___ portion(s) per day/week.

- One portion of meat is about the size of a deck of cards or the palm of your hand.
- Avoid **deli meat** (sausage, salami, pepperoni, ham, cold cuts) and **pre-prepared meat** (chicken or fish nuggets and fingers, meat pies, pâtés, hot dogs, burgers) because they often contains potassium additives.

☐ Reduce your consumption of **dairy product** to ___ portion(s) per day.

- One portion equals to ½ cup (125ml) of **milk** or **yogurt**.

☐ Avoid **processed potatoes** such as **chips and French fries**.

☐ **Boil potatoes twice** and discard the cooking water.

☐ Replace **fruit juice, sweet beverages and energy drinks** with water.

☐ Limit your **coffee** consumption to ___ cup(s) per day.

☐ Reduce your **alcohol** consumption ___ drink(s) per week.

☐ Eat less **chocolate**, **chocolate desserts**, **maple syrup** and **molasses**.

Question: Why is it important to reduce your potassium blood levels?

Answer: High blood potassium levels can lead to an abnormal heart rhythm. Very high blood potassium levels can lead to death.

Question: What are the symptoms of high blood potassium levels?

Answer: You can feel weak, have muscle weakness or suddenly be unable to walk, but often, there are **no symptoms.**

Question: What is a high level of potassium?

Answer: Blood potassium is often considered high above 5.5 mmol/L. Blood levels above 6.0 mmol/L is often considered very high.

Question: Why is the potassium in meat and milk more dangerous than the potassium in fruits and vegetables?

Answer: Potassium in meat and dairy is more readily absorbed by the body while the potassium from fruits and vegetables is only absorb up to 50 to 60%. The potassium from additives is fully (100%) absorbed by the body.

Question: Why should I avoid fruit juices more than whole fruits?

Answer: Potassium from liquid foods is more readily absorb by the body than potassium from whole foods.

References

1. Kovesdy CP, Regidor DL, Mehrotra R, et al. Serum and dialysate potassium concentrations and survival in hemodialysis patients. *Clin J Am Soc Nephrol* 2007; 2: 999-1007.
2. Kim T, Rhee CM, Streja E, et al. Racial and Ethnic Differences in Mortality Associated with Serum Potassium in a Large Hemodialysis Cohort. *Am J Nephrol* 2017; 45: 509-521.
3. Unruh ML, Evans IV, Fink NE, et al. Skipped Treatments, Markers of Nutritional Nonadherence, and Survival Among Incident Hemodialysis Patients. *Am J Kidney Dis* 2005; 46: 1107-1116.
4. Karaboyas A, Robinson BM, James G, et al. Hyperkalemia excursions are associated with an increased risk of mortality and hospitalizations in hemodialysis patients. *Clin Kidney J* 2020. DOI: 10.1093/ckj/sfaa208.
5. Pun PH and Middleton JP. Dialysate Potassium, Dialysate Magnesium, and Hemodialysis Risk. *J Am Soc Nephrol* 2017; 28: 3441-3451. DOI: 10.1681/asn.2017060640.
6. Harel Z, Harel S, Shah PS, et al. Gastrointestinal Adverse Events with Sodium Polystyrene Sulfonate (Kayexalate) Use: A Systematic Review. *The American Journal of Medicine* 2013; 126: 264.e269-264.e224. DOI: 10.1016/j.amjmed.2012.08.016.
7. Ju A, Teixeira-Pinto A, Tong A, et al. Validation of a Core Patient-Reported Outcome Measure for Fatigue in Patients Receiving Hemodialysis. *Clin J Am Soc Nephrol* 2020; 15: 1614-1621. DOI: 10.2215/cjn.05880420.
8. Kalantar-Zadeh K, Gutekunst L, Mehrotra R, et al. Understanding sources of dietary phosphorus in the treatment of patients with chronic kidney disease. *Clinical Journal of The American Society of Nephrology: CJASN* 2010; 5: 519-530. Research Support, N.I.H., Extramural Review. DOI: https://dx.doi.org/10.2215/CJN.06080809.

9. Holden RM, Mustafa RA, Alexander RT, et al. Canadian Society of Nephrology Commentary on the Kidney Disease Improving Global Outcomes 2017 Clinical Practice Guideline Update for the Diagnosis, Evaluation, Prevention, and Treatment of Chronic Kidney Disease-Mineral and Bone. *Canadian Journal of Kidney Health and Disease* 2020; 7: 205435812094427. DOI: 10.1177/2054358120944271.
10. Karp H, Ekholm P, Kemi V, et al. Differences Among Total and In Vitro Digestible Phosphorus Content of Plant Foods and Beverages. *J Ren Nutr* 2012; 22: 416-422. DOI: 10.1053/j.jrn.2011.04.004.
11. St-Jules DE, Jagannathan R, Gutekunst L, et al. Examining the Proportion of Dietary Phosphorus From Plants, Animals, and Food Additives Excreted in Urine. *J Ren Nutr* 2017; 27: 78-83. DOI: 10.1053/j.jrn.2016.09.003.
12. Carrero JJ, González-Ortiz A, Avesani CM, et al. Plant-based diets to manage the risks and complications of chronic kidney disease. *Nature Reviews Nephrology* 2020; 16: 525-542. DOI: 10.1038/s41581-020-0297-2.
13. Goraya N, Simoni J, Jo C-H, et al. Treatment of metabolic acidosis in patients with stage 3 chronic kidney disease with fruits and vegetables or oral bicarbonate reduces urine angiotensinogen and preserves glomerular filtration rate. *Kidney Int* 2014; 86: 1031-1038. 10.1038/ki.2014.83. DOI: papers3://publication/doi/10.1038/ki.2014.83.
14. Wilson J, Shah T and Nissenson AR. Hypertension in hemodialysis patients: Role of Sodium and Volume in the Pathogenesis of Hypertension in Hemodialysis. *Semin Dial* 2004; 17: 260-264. DOI: 10.1111/j.0894-0959.2004.17323.x.

15. Ikizler TA, Burrowes JD, Byham-Gray LD, et al. KDOQI Clinical Practice Guideline for Nutrition in CKD: 2020 Update. *Am J Kidney Dis* 2020; 76: S1-S107. DOI: 10.1053/j.ajkd.2020.05.006.
16. Goraya N, Simoni J, Jo C-H, et al. A comparison of treating metabolic acidosis in CKD stage 4 hypertensive kidney disease with fruits and vegetables or sodium bicarbonate. *Clin J Am Soc Nephrol* 2013; 8: 371-381. 10.2215/CJN.02430312. DOI: papers3://publication/doi/10.2215/CJN.02430312.
17. Adeva MM and Souto G. Diet-induced metabolic acidosis. *Clin Nutr* 2011; 30: 416-421. 2011/04/13. DOI: 10.1016/j.clnu.2011.03.008.
18. Grunert KG, Dean M, Raats MM, et al. A measure of satisfaction with food-related life. *Appetite* 2007; 49: 486-493. DOI: 10.1016/j.appet.2007.03.010.
